# Learning reduces ingroup bias more with perceived losses than gains across cultures

**DOI:** 10.1038/s41539-025-00362-x

**Published:** 2025-11-10

**Authors:** Yuqing Zhou, Björn Lindström, Alexander Soutschek, Pyungwon Kang, Shihui Han, Philippe N. Tobler, Grit Hein

**Affiliations:** 1 State Key Laboratory of Cognitive Science and Mental Health, Institute of Psychology, Chinese Academy of Sciences, Beijing, China; 2 Translational Social Neuroscience Unit, Department of Psychiatry, Psychosomatics, and Psychotherapy, University of Würzburg, Würzburg, Germany; 3 Karolinska Institutet, Department of Clinical Neuroscience, Solna, Sweden; 4 Department of Psychology, Ludwig Maximilian University, Munich, Germany; 5 Department of Economics and Laboratory for Social and Neural Systems Research, University of Zurich and Neuroscience Center Zurich, University of Zurich and Swiss Federal Institute of Technology Zurich, Zurich, Switzerland; 6 School of Psychological and Cognitive Sciences, Beijing Key Laboratory of Behavior and Mental Health, PKU-IDG/McGovern Institute for Brain Research, Peking University, Beijing, China

**Keywords:** Psychology, Human behaviour

## Abstract

Cultural background shapes intergroup impressions. While previous evidence suggests collectivistic cultures show stronger ingroup bias, cultural effects on impression formation processes remain unexplored. Here, we used reinforcement learning models to examine changes in intergroup impressions within gain and loss frames across individualistic (Western) and collectivistic (East Asian) cultures. Participants interacted with ingroup or outgroup individuals who increased (Gain) or decreased (Loss) their earnings, with identical net outcomes. Impression ratings were taken pre- and post-interaction. Results revealed higher ingroup identification in East Asians and initial ingroup bias in both cultures. Westerners learned to reduce this initial ingroup bias based on a learning signal (negative prediction error) generated if an ingroup individual reduced their earnings (i.e., Loss frame). East Asians showed the same learning mechanism, but only with low ingroup identification. Together, we show that learning from negatively perceived ingroup interactions can decrease ingroup bias across cultures, modulated by individual ingroup identification.

## Introduction

Ample evidence indicates that individuals typically form more positive impressions of ingroup members than outgroup members^1–3^. While this bias can strengthen social cohesion within groups (i.e., ingroup love, Everett et al.^4^, it often promotes negative evaluations and discrimination toward outgroups (i.e., outgroup hate, Halevy et al.)^5^, escalating intergroup conflict.

Although this ingroup bias occurs across cultures^6^, it tends to be stronger in collectivistic societies compared to individualistic ones^7–9^. This cultural variation likely arises from psychological differences in group loyalty and identity between collectivism and individualism. Specifically, individualists generally exhibit less group loyalty and prioritize personal goals over collective ones, whereas collectivists typically align their personal goals with collective goals or subordinate them entirely^10–12^. Consistent with these predictions, empirical research confirms that individuals in collectivist cultures show stronger group identification than those in individualistic cultures^9^. That said, it is important to notice that individualism and collectivism should not be seen as polar opposites^11,13,14^. Independently of culture, each individual carries both individualistic and collectivist tendencies and could behave in an individualistic and collectivistic manner^15–18^. The difference is that in some cultures (i.e., Western cultures) the probability of situations fostering individualistic tendencies is high, while other cultures (i.e., East Asian cultures) are more likely to foster collectivistic tendencies. Thus, on top of individual differences, culture is a factor that strongly modulates collectivism and individualism and associated ingroup identification.

Culture-induced differences in ingroup identification are important, because they are linked to intergroup impressions^19^. Biased ingroup impressions, in turn, are an important determinant of human behavior in politics^20^, law enforcement^21^, financial markets^22^, and employment^23^. Therefore, the question of how to decrease such ingroup bias in both individualistic and collectivistic cultures is of high practical relevance.

A core mechanism of impression formation is learning. Drawing on learning theory, impressions are continuously updated through experiences: direct interactions form associations with individuals, where positive experiences foster positive associations and negative experiences produce negative ones^24–27^. Recent evidence extends this process to observational learning, showing that witnessing others’ actions similarly shapes impressions via vicarious learning^28^. These updates are driven by prediction errors—discrepancies between expected and actual outcomes^29–34^. Impression updating thus involves gradually reducing prediction errors by adjusting expectations about individuals or groups. The rate of updating is not fixed but can be modulated by the uncertainty of prior beliefs. For example, higher prior uncertainty accelerates updating in response to prediction errors. Conversely, strong prior beliefs can lead to slower updating when encountering counter-attitudinal information, potentially as individuals discount prediction errors by inferring alternate causes for unexpected behavior^35–37^. This framework enables the formal testing of specific learning patterns through computational modeling^38^.

Building on this learning framework, recent research shows that social group information significantly influences impression formation, though findings are inconclusive. On the one hand, studies indicate individuals form more positive impressions of ingroup partners than outgroup partners despite identical objective experiences (e.g., sharing money). Computational reinforcement learning models reveal that this persistent ingroup bias stems from people learning more readily about negative outgroup behavior^24,26,27,39^. On the other hand, other evidence demonstrates that learning can reduce biased ingroup impressions. Specifically, learning from identical experiences with both groups (e.g., relief from pain) significantly decreased ingroup bias in impression ratings. Mechanistically, reinforcement learning modeling showed that unexpected negative ingroup experiences generated a learning signal (prediction error) by conflicting with participants’ positive ingroup expectations, accounting for this reduction in bias^34^. Synthesizing these findings highlights perceived valence of social interactions as a critical factor: studies showing persistent ingroup bias typically involve positively framed interactions (e.g., gaining rewards), while those showing reduced bias often involve negatively framed interactions (e.g., experiencing losses such as receiving shocks).

This critical role of perceived valence—specifically the distinct impact of losses and gains—finds parallel support in broader learning research. In greater detail, learning studies outside the social domain have shown that individuals learn differently from perceived losses than from perceived gains. For example, early studies showed that participants learned faster when minimizing loss compared to when seeking gain^40,41^. This asymmetry in learning is typically explained by loss aversion, reflecting the notion that losses loom larger than gains of the same objective magnitude, facilitating the selection of behavioral strategies that minimize losses^42,43^. Studies focusing on intergroup interactions but not on learning suggest that ingroup bias in the gain domain (when giving to others) is smaller or even absent in the loss domain (when taking from others)^44,45^. Some recent evidence also showed that framing a choice as preventing others’ monetary loss instead of raising others’ gains increased allocations to socially distant others^46^.

Integrating these different lines of research, we reasoned that the extent of learning-related changes in intergroup impressions depends on whether ingroup and outgroup interactions are perceived as a gain or as a loss. In addition, given that collectivistic cultures show higher tolerance to negative experiences generated by ingroups^47,48^, individuals from such cultures might be less sensitive to negative ingroup experiences and show less learning from these experiences, reflecting distinct social learning in the intergroup context between cultures. Clarifying these framing effects is important to understand the formation and alteration of intergroup impressions in different cultures. However, so far it is unclear whether individuals learn to change intergroup impressions differently in a gain and a loss frame and how these effects are shaped by culture.

**Fig. 1:**
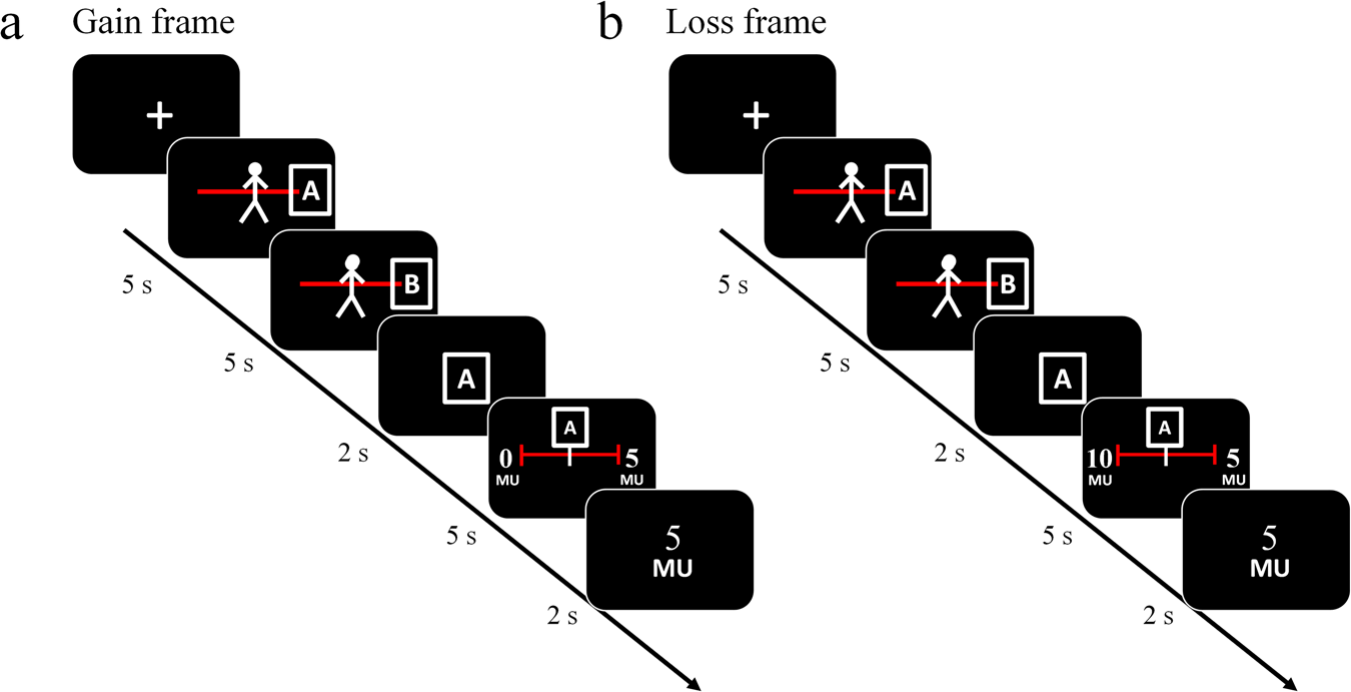
Example trial sequence. At the beginning of each trial, the Western (Study 1) or East Asian (Study 2) participants rated their closeness toward the ingroup and outgroup on separate rating scales that were presented in randomized order. To do so, they moved a manikin representing themselves toward or away from an ingroup and an outgroup “room”, denoted as rooms “A” and “B” in counterbalanced order. The next screen revealed the room in which the ostensible other player in this round was sitting (i.e., the ingroup or the outgroup room). Participants then indicated their expectancy of receiving 5 MU (vs. 0 MU, Gain frame, panel **a**) or losing 5 MU (vs. 10 MU, Loss frame, panel **b**) to the other person in the current trial using a 10-step rating scale. In the Gain frame, participants received no initial endowment, and the other player could choose to give zero or five MU. In the Loss frame, participants received an initial endowment of ten MU, and the other player could choose to take ten or five MU away from the participant. The two frames were mathematically equivalent, i.e., they yielded identical final payoff states to the participant and the other person. MU refers to CHF in Study 1 and RMB in Study 2, representing the local currency.

Here, we use reinforcement learning, computational modeling, and a cross-cultural design to test how ingroup and outgroup experiences in Gain and Loss frames affect dynamic changes in ingroup and outgroup closeness and impressions across cultures. In our study, Swiss (Study 1) and Chinese (Study 2) participants repeatedly interacted with one of several individuals from an ingroup or an outgroup (confederates). Participants were randomly assigned to either a Gain frame (where they could be given money from others) or a Loss frame (where money could be taken away from them). Crucially, both frames resulted in identical final payoffs for both the participant and the others (see Methods for details). During the experiment, participants rated their perceived closeness to ingroup and outgroup members, their expectancy of receiving or losing money in each trial, and their impressions of both groups before and after the task (Fig. 1). This design enabled us to model how participants learned from equivalent ingroup and outgroup experiences and how these learning processes shaped intergroup impressions. Specifically, we employed computational reinforcement learning models to elucidate the mechanisms underlying the change in intergroup impressions, estimate the learning parameters that capture trial-to-trial changes in expectancy and closeness ratings, and link them to impression change.

Based on previous evidence, we hypothesized that initially, both samples would show more positive impressions towards the ingroup compared to the outgroup. Given the evidence for stronger ingroup identification^47,49^ and ingroup biases^8^ in East Asian than Western samples, one might expect ingroup identification scores and the ingroup bias in impression ratings to be larger in East Asian compared to Western participants. According to the frame-independent hypothesis, equal experiences with both groups may reduce intergroup bias in impression differences across Gain and Loss frames^34,50,51^. This occurs because such experiences conflict with participants’ negative outgroup and positive ingroup expectations, generating positive outgroup and negative ingroup prediction errors. Alternatively, learning- related intergroup impression changes might differ in the Gain and Loss frames due to two interacting mechanisms. First, loss aversion—the tendency for losses to exert greater impact than equivalent gains^52^—implies that participants in the Loss frame exhibit heightened sensitivity to unexpected negative outcomes. Second, individuals typically hold positive prior expectations about ingroups^53^. When ingroup members violate these expectations, the resulting negative prediction errors are larger than the prediction errors arising from equivalent violations by outgroups. This combination suggests that the Loss frame may reduce ingroup bias more strongly than the Gain frame. In East Asian cultures, where ingroup identification is typically stronger^49^, individuals may hold even more positive prior expectations of ingroups. This may amplify both negative ingroup prediction errors (e.g., greater surprise at ingroup harm) and positive outgroup prediction errors (e.g., unexpected cooperation from outgroups), resulting in greater learning-related changes in closeness and intergroup impressions. Alternatively, the experience may not provide enough evidence, and strong positive prior expectations of ingroups may prevent learning the negative events generated by ingroups^36^. This would be in line with previous studies showing that people are less influenced by their social partners’ behavior when the behavior is inconsistent with their prior impressions of their partners^54^.

## Results

### Manipulation checks across studies

If our group manipulation was successful, we should observe ingroup biases in expectancy, closeness and impression ratings. To validate the effect of the group manipulation, we ran linear mixed models (LMM) with frame (Gain/Loss), group (ingroup/outgroup), culture (Western/East Asian) and their interaction terms as predictors, and with the initial impression rating, expectancy ratings (first trial), and closeness ratings (first trial) as dependent variables. For all three measures, we found a significant effect of group. Specifically, initial impressions were more positive (χ2 = 176.28, *p* < 0.001, *β* = 0.389, 95% CI = [0.33 0.45]) and initial expectations (χ2 = 9.20, *p* = 0.002, *β* = 0.129, 95% CI = [0.045 0.212]), as well as initial closeness (χ2 = 45.11, *p* < 0.001, *β* = 0.234, 95% CI = [0.165 0.303]) were higher for the ingroup (Table 1 for descriptives; Supplementary Table 1 for full statistical details).

**Table 1:** Comparison of impression, closeness, expectations, and ingroup identification ratings between the Western (Study 1) and the East Asian (Study 2) sample

Variables	Western sample (Study 1, *N* = 112)	East Asian sample (Study 2, *N* = 100)	T-test		
	Mean ± SD	Mean ± SD	T-value	*p*	Cohen’s d
Ingroup impression	5.99 ± 1.12	6.69 ± 1.14	4.59	<0.001	0.62
Outgroup impression	5.68 ± 1.14	5.04 ± 1.08	−4.14	<0.001	0.58
Ingroup closeness	4.24 ± 2.43	7.04 ± 2.03	8.00	<0.001	1.25
Outgroup closeness	4.76 ± 2.41	4.17 ± 2.16	−1.80	0.069	0.25
Ingroup expectation	0.63 ± 0.36	0.57 ± 0.34	−1.32	0.189	0.17
Outgroup expectation	0.57 ± 0.37	0.44 ± 0.33	−2.77	0.006	0.37
Ingroup identification	4.57 ± 1.15	5.75 ± 0.91	8.93	<0.001	1.26

The group×culture interactions were significant for impression and closeness ratings (impression/closeness: χ2 = 83.39/92.36, *ps* < 0.001, *β* = 0.268/0.335, 95% CI = [0.210 0.325]/[0.266 0.404]; Supplementary Table 1 for full statistical details), reflecting a stronger initial ingroup bias in the East Asian compared to the Western sample. Post hoc t-tests showed that compared to Western participants, East Asian participants felt significantly closer to and had better impressions of the ingroup and worse impressions of the outgroup. Moreover, East Asian participants identified significantly more strongly with the ingroup compared to Western participants (Table 1).

In both samples, there was a significant positive relationship between ingroup identification scores and the initial ingroup bias (ingroup vs. outgroup difference) in impression ratings (Western sample: *r* = 0.26, *p* = 0.009; East Asian sample: *r* = 0.25, *p* = 0.011), suggesting that higher ingroup identification was associated with greater ingroup bias at the beginning of the experiment.

### Greater change of intergroup impressions in the loss frame compared to the gain frame (Study 1, Western sample)

**Fig. 2:**
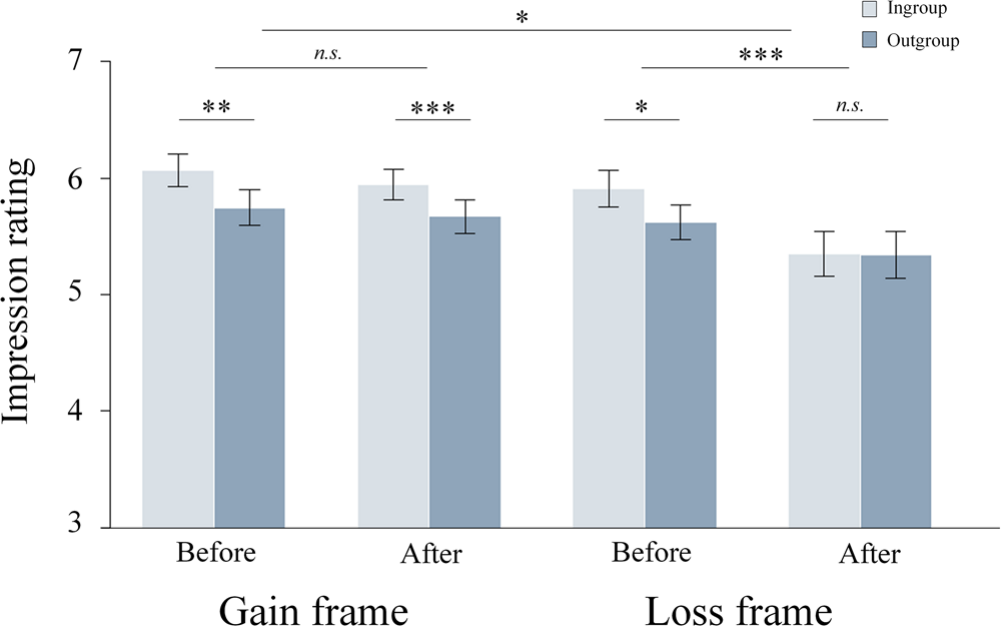
Ingroup and outgroup impressions before and after learning in the Western sample. Learning reduced the significant initial ingroup bias in the Loss frame, but not in the Gain frame. Larger values represent more positive impressions. ***p < 0.001, **p < 0.01, *p < 0.05.

Focusing first on Study 1, we investigated whether participants changed the reported initial ingroup biases and whether the effects differed in the Gain and Loss frame. An LMM with frame (Gain/Loss), group (ingroup/outgroup), time (before/after learning), and their interaction terms as predictors, and the impression rating scores as the dependent variable revealed a significant frame × group × time interaction (*χ*2 = 4.78, *p* = 0.029, *β* = −0.024, SE = 0.011, 95% CI = [-0.05 -0.002], Fig. 2). To unpack this interaction effect, we conducted separate LMMs for the Gain frame and Loss frame. In the Gain frame, the group × time interaction was not significant (*χ*2 = 0.42, *p* = 0.52, *β* = 0.01, *SE* = 0.016, 95% CI = [-0.02 0.042], Fig. 2), reflecting a similar ingroup bias in impression ratings before learning (*χ*2 = 9.01, *p* = 0.003, *β* = −0.14, *SE* = 0.047, 95% CI = [-0.24 -0.05]) and after learning (*χ*2 = 11.5, *p* < 0.001, *β* = −0.13, *SE* = 0.038, 95% CI = [-0.21 -0.05]). Analyzing the impression changes separately for the ingroup and the outgroup revealed no significant pre-to-post learning changes: ingroup (χ2 = 1.89, *p* = 0.17, *β* = −0.057, SE = 0.042, 95% CI = [-0.14 0.03]); outgroup (χ2 = 1.12, *p* = 0.29, *β* = −0.033, SE = 0.031, 95% CI = [-0.10 0.03]). By contrast, in the Loss frame, we observed a significant group × time interaction (*χ*2 = 11.5, *p* < 0.001, *β* = 0.09, *SE* = 0.027, 95% CI = [0.038 0.15] Fig. 2). In follow-up analyses, we compared ingroup vs. outgroup impressions separately before and after learning. The results showed significantly more positive impressions towards the ingroup compared to outgroup members before learning (*χ*2 = 3.99, *p* = 0.046, *β* = −0.127, *SE* = 0.064, 95% CI = [-0.25 0.00]), but no significant difference between groups after learning (*χ*2 = 0.01, *p* = 0.93, *β* = −0.004, *SE* = 0.042, 95% CI = [-0.088 0.080]).

Analyzing the impression changes separately for the ingroup and the outgroup revealed a significant reduction of ingroup impressions (χ2 = 13.1, *p* < 0.001, *β* = −0.56, SE = 0.15, 95% CI = [-0.87 0.25]) while outgroup impressions did not change significantly (χ2 = 3.65, *p* = 0.056, *β* = −0.11, SE = 0.056, 95% CI = [-0.22 0.01]). Thus, framing influenced the change of intergroup impressions, and in particular, ingroup impressions. After objectively similar experiences caused by ingroup and outgroup members, participants maintained the ingroup bias in the Gain frame but decreased it in the Loss frame.

### Changes of intergroup impressions in the Loss frame are related to a stronger weight of negative ingroup prediction errors (Study 1, Western sample)

To investigate the mechanisms driving the shifts in intergroup impressions observed in Fig. 2, we employed computational reinforcement learning (RL) models. These models formalize how participants update their expectancy and closeness ratings in response to prediction errors—discrepancies between expected and actual outcomes. Specifically, trial-to-trial changes in ratings were modeled as a function of prediction errors, with learning parameters quantifying the extent to which these errors influenced updates to expectancy and closeness (e.g., higher parameter values indicate stronger reliance on unexpected new outcomes).

After estimating these learning parameters, we conducted multiple linear regression analyses to evaluate their role in reducing intergroup impression bias (i.e., the interaction effect in Fig. 2). Here, the learning parameters served as predictors, while changes in intergroup impression ratings (pre- vs. post-learning) acted as dependent variables. This approach allowed us to disentangle how individual differences in learning dynamics contributed to the attenuation of bias.

**Fig. 3:**
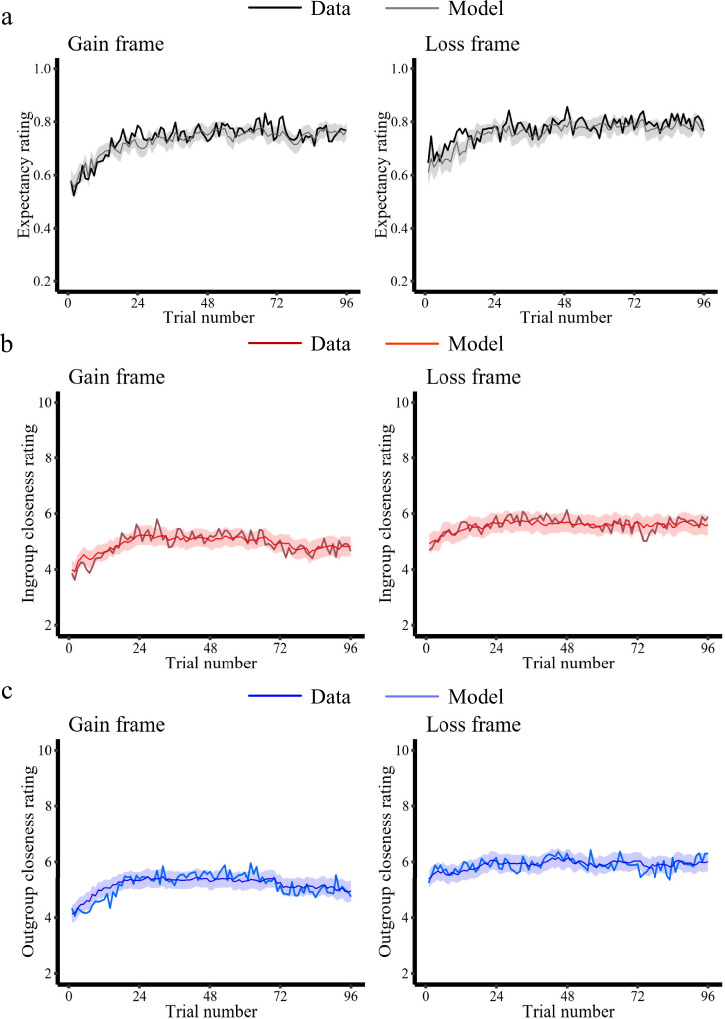
Computational models explain expectancy ratings and in- and outgroup closeness ratings in the Western sample. **a** Predictions of money being given/taken varied over the course of the experiment (solid black line) and our learning model explained these predictions (shaded area). **b** Trial-by-trial ingroup closeness ratings (solid red line) and corresponding model estimates (shaded area) in the Gain and Loss frame. **c** Trial-by-trial outgroup closeness ratings (solid blue line) and corresponding model estimates (shaded area) in the gain and loss frame. The model estimates illustrate the best-fitting Learning/Closeness model. All panels show averages of participants (*N* = 58/54 in the Gain/Loss frame). The shaded area represents$\pm$ s.e.m. around the mean model predictions.

First, we modeled participants’ trial-by-trial expectancy ratings using a Rescorla-Wagner reinforcement-learning model (Rescorla & Wagner, 1972) to test for differences in basic learning mechanisms between the ingroup and outgroup conditions (Eqs. 1–3, see Methods for details). We defined reinforcement learning models in which the initial expectancies were based on the first trial of the actual data. This approach has been used in our previous research^34,50^ to capture individual variability in initial expectations, thereby enhancing the model’s fit. We first verified that our candidate models were accurately recovered by our estimation approach, which ensures a meaningful comparison of different learning models (Supplementary Fig. 1A). The model comparison revealed that, for both Gain frame and Loss frame, expectancy ratings were best characterized by a model with different learning rates for negative and positive prediction errors generated by the ingroup and the outgroup (Learning Model 3: Loss frame: *r*^2^ = 0.33 ± 0.26; Gain frame: *r*^2^ = 0.38 ± 0.30; mean ± SD; Supplementary Fig. 1C and Fig. 3a). Independent parameter recovery analyses using the winning learning model with simulated data showed good recovery over a wide parameter space (*rs* > 0.88, Supplementary Fig. 1B). We then extracted the estimated model parameters from the winning reinforcement learning model and performed an LMM with frame (Gain/Loss), group (ingroup/outgroup), valence (positive/negative), and their interaction terms as predictors, and the learning rate as the dependent variable. The results only showed a significant main effect of valence as the learning rate for the positive prediction errors was larger than for negative prediction errors (*χ*2 = 112.97, *p* < 0.001, *β* = −0.52, *SE* = 0.049, 95% CI = [-0.62 -0.43]). No other significant effects were found in this model (*ps* > 0.14; see Supplementary Table 2 and Supplementary Table 3 for details). Together, these results demonstrate that participants’ trial-by-trial expectancy ratings towards an ingroup and an outgroup are dynamically updated based on the positive and negative prediction errors elicited by the behavior of in- and outgroup members.

Second, we tested how ingroup and outgroup prediction errors affected trial-by-trial changes in ingroup and outgroup closeness ratings. To do so, we modeled the trial-by-trial update of in- and outgroup closeness ratings as a linear function of the cumulative impact of prediction errors, which we estimated with the best-fitting reinforcement learning model. We initially verified good model recovery concerning our main model space, which allowed us to compare the different closeness models (Supplementary Fig. 1A) meaningfully. The winning model (Eq. 5, Closeness Model 2) successfully captured dynamic changes of closeness ratings at the individual level for both the ingroup (Loss frame: *r*^*2*^ = 0.28 ± 0.24; Gain frame: *r*^*2*^ = 0.30 ± 0.28; mean ± SD; Supplementary Fig. 1D and Fig. 3b) and the outgroup (Loss frame: *r*^*2*^ = 0.26 ± 0.21; Gain frame: *r*^*2*^ = 0.29 ± 0.26; Supplementary Fig. 1D and Fig. 3c). The winning model assumes that trial-wise closeness ratings related to a particular group are driven by the time-discounted sum of previous prediction errors by adding up separately modeled positive and negative prediction errors (see *Methods* for additional details). Analyses of simulated data showed good parameter recovery (*rs* > 0.98, Supplementary Fig. 1B) for the winning closeness model over a wide parameter space.

We also fitted the changes in ingroup and outgroup closeness ratings separately and estimated group-specific parameters for the two frames. We compared the weight parameters between the Gain and Loss frames but found no significant differences (*ps* > 0.13; see Supplementary Table 3 for full statistical results). Together, these results demonstrate that ingroup and outgroup closeness is dynamically updated based on prediction errors elicited by the behavior of in- and outgroup members.

**Fig. 4:**
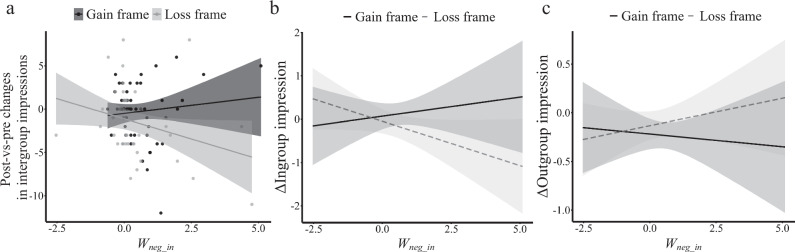
Linking model parameters to post- vs. pre-learning changes in intergroup impression in the Western sample. **a** Correlation between the individual weight of negative ingroup prediction errors (*W*_*neg_in*_) and the post-vs. pre-learning changes in intergroup impressions in the Gain and Loss frame. These changes represent the decrease of ingroup bias in impressions after learning, that is, (ingroup-outgroup)_after_ – (ingroup-outgroup)_before_. Smaller values indicate greater decreases of ingroup bias. **b** Framing moderated the association between the *W*_*neg_in*_ parameter and the learning-induced change in ingroup impression. **c** Framing did not moderate the association between the *W*_*neg_in*_ parameter and the change of outgroup impression.

Third, we determined the learning parameters that account for the observed change in intergroup impressions (Fig. 2). We first conducted a stepwise multiple linear regression analysis explaining the post- vs pre-learning differences in intergroup impressions (ingroup vs outgroup). As independent variables, we used the *weight* and *learning rate* parameters, the key parameters governing learning. In the Loss frame, the stepwise regression revealed only a significant effect of the weight given to the negative ingroup prediction errors, i.e., the *W*_*neg_in*_ parameter (*β* = −0.13, *t(52)* = −2.0, *p* = 0.05, Fig. 4a, Spearman correlation: *rho* = −0.37, *p* = 0.013). Deleting outliers and influencing points did not change the association (*t(46)* = −2.51, *p* = 0.016). We then examined the relationship between *W*_*neg_in*_ parameter and ingroup/outgroup impression changes separately. The results revealed that the *W*_*neg_in*_ parameter specifically predicted the change in ingroup impression (rho = −0.36, *p* = 0.011) but not outgroup impression (rho = −0.16, *p* = 0.27). By contrast, the *W*_*neg_in*_ parameter did not significantly predict the post- vs. pre-learning differences in intergroup impressions for the Gain frame (*β* = 0.06, *t(56)* = 0.80, *p* = 0.43, Fig. 4a). Our findings indicate that participants who put a stronger weight on negative ingroup events to update social closeness in the Loss frame also showed a greater reduction in ingroup bias in impression. This relationship was absent in the Gain frame.

To formally compare the relationship between *W*_*neg_in*_ and the intergroup impression change in the Gain and Loss frame, we conducted a moderation analysis with frame (gain/loss) as the moderator, *W*_*neg_in*_ served as the independent variable, and post-vs-pre changes in intergroup impressions (ingroup vs. outgroup) as the dependent variable. We found that the relationship between the *W*_*neg_in*_ parameter and the post-vs-pre changes in intergroup impression ratings was marginally moderated by the frame (*β* = 0.20, *t(108)* = 1.91, *p* = 0.059). We also examined the moderation effect separately for the change of ingroup and outgroup impression, with the change of outgroup or ingroup impression as the control variable, respectively. The results showed that framing significantly moderated the relationship between *W*_*neg_in*_ and changes in ingroup impressions (i.e., the decrease in ingroup impression *β* = −0.23, *t(107)* = −2.32, *p* = 0.022, Fig. 4b), but not the relationship between *W*_*neg_in*_ and the change in outgroup impression (*β* = 0.08, *t(107)* = 0.85, *p* = 0.40, Fig. 4c). Taken together, our results suggest that the objectively same experiences with the ingroup and outgroup decreased ingroup bias in impression rating in the Loss frame but not in the Gain frame. The differential changes in intergroup impression between frames were accompanied by the unique relationship between the *W*_*neg_in*_ parameter and the decrease of ingroup impression in the Loss frame.

### Greater change of intergroup impressions in the loss frame compared to the Gain frame, but only if ingroup identification is low (Study 2, East Asian sample)

Study 1 (Western sample) revealed that framing identical outcomes as losses rather than gains decreases the ingroup bias in impression ratings. Moreover, learning from unexpected negative ingroup experiences in the Loss frame (i.e., *W*_*neg_in*_) uniquely contributed to the decrease of ingroup impression. In Study 2, we tested whether these effects generalized to a sample from a more collectivistic culture characterized by stronger ingroup identification and ingroup bias (Table 1).

**Fig. 5:**
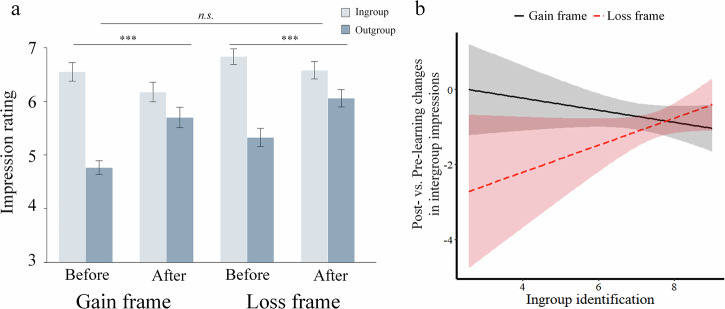
Ingroup and outgroup impressions before and after learning in the East Asian sample. **a** On average, the decrease of ingroup bias did not differ between the Gain and the Loss frame. Larger values represent more positive impressions. **b** Different relationships between scores on the ingroup identification scale and post- vs. pre-learning changes in intergroup impressions (ingroup vs. outgroup) in the Gain and the Loss frame. Smaller values indicate greater decreases of ingroup bias in impression ratings. ***p < 0.001, **p < 0.01, *p < 0.05.

We first sought to compare the results between the Western and East Asian sample. To this end, we ran an LMM with frame (gain/loss), group (ingroup/outgroup), time (before/after learning), culture (East Asian/Western) and their interaction terms as predictors, and the impression rating scores as the dependent variable. The culture × frame × group × time interaction revealed a non-significant trend of weaker frame × group × time interaction for East Asian participants compared to Western participants (χ2 = 2.96, *p* = 0.085). Unlike the Western participants, East Asian participants showed no significant frame × group × time interaction (*χ*2 = 1.01, *p* = 0.31, *β* = 0.06, *SE* = 0.061, 95% CI = [-0.06 0.18], Fig. 5a). Then, we ran an LMM with frame (gain/loss), group (ingroup/outgroup), time (before/after learning), and their interaction terms as predictors, and the impression rating scores as the dependent variable. The group × time interaction was significant (*χ*2 = 50.15, *p* < 0.001, *β* = −0.35, *SE* = 0.061, 95% CI = [-0.47 -0.23]). Analyzing the impression changes separately for the ingroup and the outgroup showed that learning improved outgroup impressions (χ2 = 29.62, *p* < 0.001, *β* = 0.38, SE = 0.070, 95% CI = [0.24 0.52]), whereas ingroup impressions became more negative (χ2 = 4.10, *p* = 0.043, *β* = −0.16, SE = 0.078, 95% CI = [-0.31 -0.003]). Further analyses showed that the change of outgroup impressions was significantly larger than the change of ingroup impressions (M(ingroup change) = 0.31 vs. M(outgroup change) = 0.83, t(99) = −2.647, *p* = 0.009). Given that East Asian participants identified more strongly with the ingroup than Western participants (*t*(203) = 8.93, *p* < 0.001, Cohen’s *d* = 1.26), it is possible that impressions held by East Asian individuals with weaker (Western-like) ingroup identification changed similarly as in the Western sample. We thus tested the influence of ingroup identification on the decrease of ingroup bias in impression ratings. To this end, we conducted a regression analysis with the post- vs. pre-learning changes in intergroup impression ratings as the dependent variable and the individual ingroup identification scores, frame (gain/loss), and their interaction term as predictors. The frame×ingroup identification interaction was significant (*β* = 0.41, *t(95)* = 2.96, *p* = 0.004, Fig. 5b). Clarifying this interaction effect, Fig. 5b shows that the less people identified with the ingroup the more strongly decreased their ingroup bias in the Loss frame, while it showed a reverse pattern in the Gain frame. In an exploratory analysis, we also checked for a modulating effect of ingroup identification in Study 1 (Western sample), without significant results (ingroup identification × frame effect: *β* = 0.26, *t(92)* = 1.23, *p* = 0.22) but a similar pattern as in the East Asian sample (Supplementary Fig. 2). Together, these results showed that for East Asian participants, framing affects intergroup impression change, but only for participants with low ingroup identification.

### Changes of intergroup impressions in the Loss frame in individuals with low ingroup identification are related to a stronger weight of negative ingroup prediction errors (Study 2, East Asian sample)

**Fig. 6:**
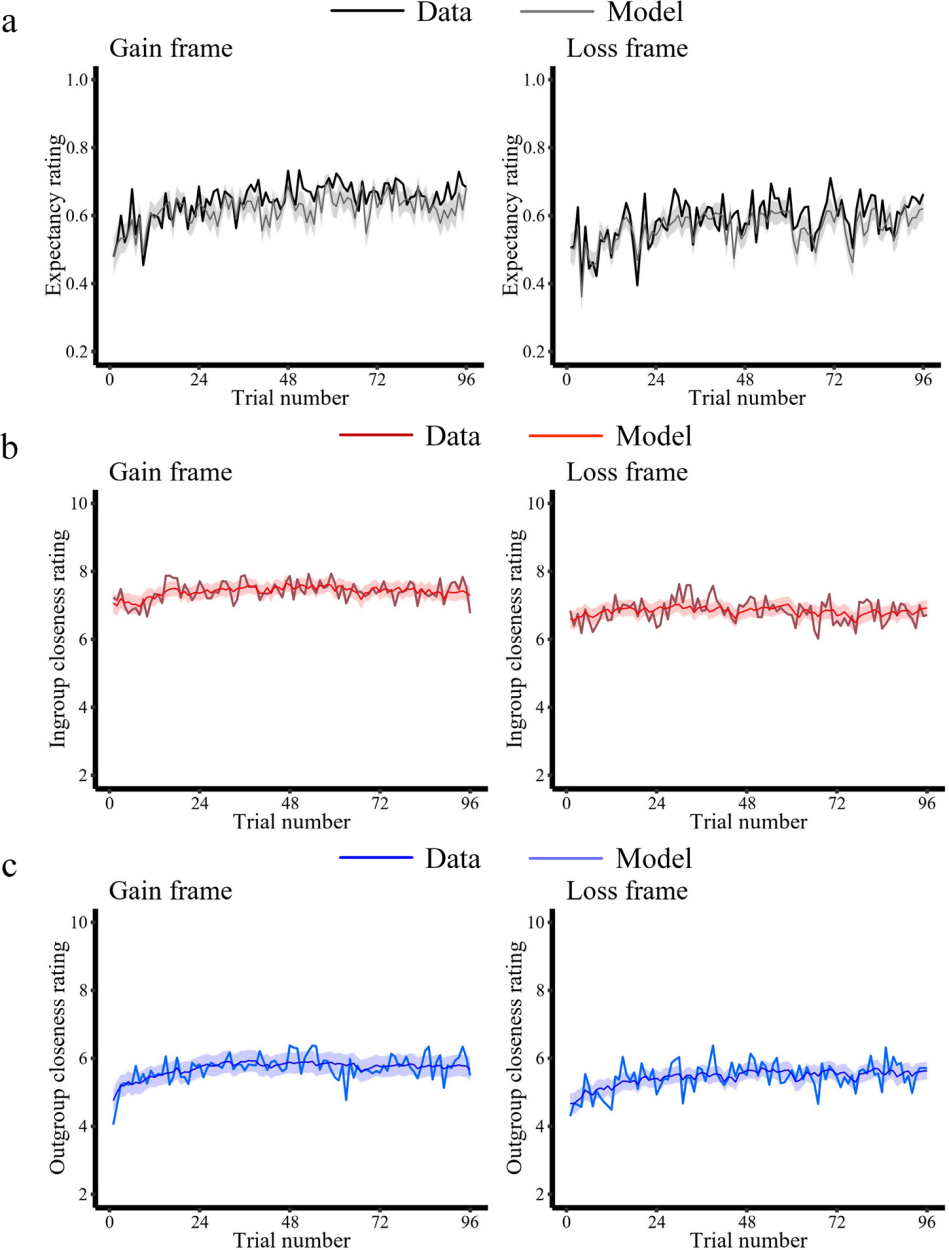
Computational models explain expectancy ratings and in- and outgroup closeness ratings in the East Asian sample. **a** Expectations of money being given/taken varied over the course of the experiment (solid black line) and our learning model explained these expectations (shaded area). **b** Trial-by-trial ingroup closeness ratings (solid red line) and corresponding model estimates (shaded area) in the Gain and Loss frame. **c** Trial-by-trial outgroup closeness ratings (solid blue line) and corresponding model estimates (shaded area) in the Gain and Loss frame. The model estimates illustrate the best-fitting Learning/Closeness model. All panels show averages of participants (*N* = 50/49 in the Gain/Loss frame). The shaded area represents $\pm$ s.e.m. around the mean model predictions.

As with Study 1, we next examined the learning process of East Asian participants, and linked learning-related parameters to the change of intergroup impressions. The model comparison also revealed that Learning Model 3 (Supplementary Table 4, Fig. 6) and Closeness Model 2 as best models for our East Asian participants (Supplementary Table 5, Fig. 6).

We again extracted the parameters from the winning models and performed LMMs with valence, group, and frame, as well as their interactions as predictors and the weight/learning rates parameters as dependent variables. As in Study 1, frame had no significant effect on both the weight and the learning rate parameters (see Supplementary Table 6 and S7 for detailed statistical information). We also found that the learning rate was modulated by a significant group×valence interaction (*χ*2 = 5.20, *p* = 0.023, *β* = −0.08, *SE* = 0.036, 95% CI = [-0.152 -0.011], Supplementary Table 6 and S7, Supplementary Fig. 3), related to the cultural differences in ingroup identification scores (see *supplementary results* for details).

**Fig. 7:**
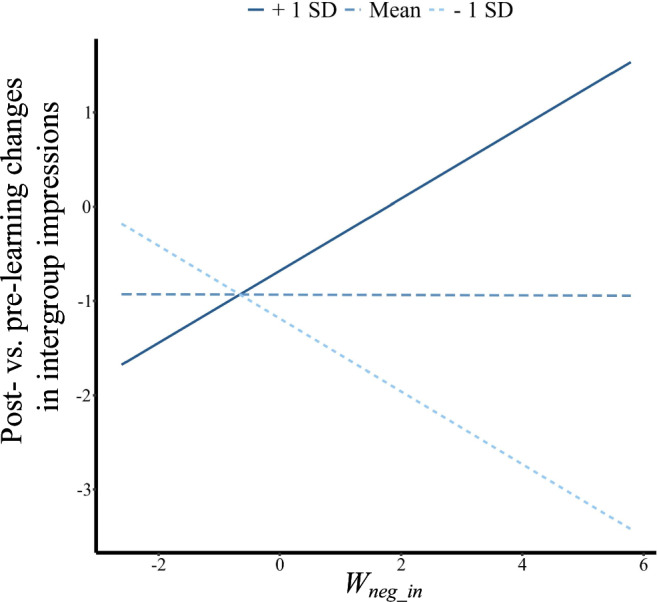
Linking model parameters to post- vs. pre-learning changes in intergroup impressions (ingroup vs. outgroup) in the East Asian sample. Ingroup identification moderated the association between the *W*_*neg_in*_ parameter (the weight given to ingroup prediction errors to update closeness ratings) and the post- vs. pre-learning changes in intergroup impressions, that is, (ingroup-outgroup)_after_ – (ingroup-outgroup)_before._ Smaller values indicate greater decreases of ingroup bias.

In Western participants, the reduction of the ingroup bias was associated with the weight of the negative ingroup prediction errors (*W*_*neg_in*_). Testing the main effect of this parameter on post-vs-pre intergroup impression change in Study 2 revealed no significant relationship (*β* = −0.028, *t(47)* = −0.21, *p* = 0.83). This would be expected given that, on average, there is no significant difference between frames on the reduction of ingroup bias after learning for the East Asian sample. However, the behavioral results reported above (Fig. 5B) showed a significant effect of ingroup identification. Inspired by this finding, we again tested the relationship between intergroup impression change and *W*_*neg_in*_ and included the individual ingroup identification scores as moderating variables. A moderation analysis with ingroup identification scores, *W*_*neg_in*,_ and their interactions as predictors and the intergroup impression change as dependent variable showed a significant moderation effect of ingroup identification (*β* = 0.50, *t(45)* = 2.97, *p* = 0.005). The lower the ingroup identification scores, the more negative was the relationship between the *W*_*neg_in*_ parameter and intergroup impression change (i.e., greater *W*_*neg_in*_ was associated with a greater decrease of ingroup bias in impression ratings, Fig. 7). Furthermore, this moderating effect was mainly driven by changes in outgroup impressions (β = −0.41, t(45) = −2.33, *p* = 0.024). We ran a similar moderation analysis on the Gain frame but found no significant moderation effect (*β* = 0.09, *t(46)* = 0.33, *p* = 0.745).

Taken together, the results in Study 2 suggest that only for those East Asian participants who identified weakly with their ingroup, the Loss frame had a stronger influence on the decrease of ingroup bias compared to the Gain frame. The decrease of ingroup bias in the Loss frame was related to learning unexpected negative outcomes drawn from the ingroup (reflected by *W*_*neg_in*_ parameters), and the coupling was strengthened when participants endorsed lower ingroup identification.

## Discussion

We show that interacting with ingroup and outgroup members with objectively the same net outcomes can dynamically reduce bias in intergroup impressions. These effects were particularly prevalent when the endowment of the participants was reduced by ingroup members (loss frame) even though the net outcomes of the participants were identical in the two frames. When participants experienced such subjective (but not objective) loss, they dynamically lowered their impression of the ingroup, which, in turn, became more equal to the impression of the outgroup. Apart from revealing the basic mechanism, conducting the same learning study in different cultures sheds additional light on learning-related intergroup impression change. Learning-related changes in intergroup impressions in the Loss frame were similar for individuals from a Western individualistic culture (Switzerland) and individuals with low ingroup identification from an East Asian collectivistic culture (China). In contrast, East Asian participants who identified strongly with their ingroup showed no reduction of ingroup impression bias, even if ingroup members reduced their endowment in the Loss frame. Notably, we observed these similarities in learning mechanisms across different cultures and different ingroup-outgroup constellations (Study 1: Swiss-Middle East; Study 2: Chinese-German; see *Supplementary methods* for details of group induction), emphasizing the robustness of the effect.

The finding that learning can change intergroup impressions with a particularly strong influence of negative ingroup experiences (weight of negative ingroup prediction errors) is in line with previous findings^34,55^. In this previous research, ingroup and outgroup members could save participants from pain, and negative ingroup prediction errors were observed when ingroup members refrained from doing so. Extending and substantiating these findings, the current results show that individuals learn more from perceived ingroup-induced losses even if the outcome is not physically harmful and, in fact, equivalent to the outcome of ingroup interactions that are perceived as gains.

Our results suggest that the learning mechanisms that shape intergroup impressions hold across cultures but can be modified by cultural differences in ingroup identification. Recent research found that Chinese, compared to American individuals, learn to punish unfair ingroup members more slowly than unfair outgroup members^47^, suggesting a higher tolerance for negative ingroup experiences in Chinese people. Echoing this recent finding, the current findings suggest that a learning-related decrease in biased impressions favoring the ingroup is more likely in individuals—and by extension, cultures—characterized by low levels of ingroup identification.

These cultural differences in identification and tolerance for ingroup transgressions led to distinct pathways for bias reduction: In the Western (Swiss) sample, reduced intergroup bias stemmed primarily from decreased ingroup love, reflected in lowered ingroup impressions. This aligns with evidence suggesting that individualistic cultures exhibit weaker constraints on ingroup loyalty, facilitating a more critical reappraisal of ingroup flaws^10–12^. Conversely, in the East Asian (Chinese) sample, bias reduction manifested predominantly through decreased outgroup hate, evidenced by elevated outgroup impressions. This pattern may arise from strong initial ingroup bias rooted in collectivist values, potentially rendering ingroup impressions more resistant to change. Furthermore, positive interactions with outgroup members may have exerted a more pronounced effect on pre-existing negative expectations, generating larger positive prediction errors and thereby significantly elevating outgroup impressions^50,51,56^. Together, this asymmetry demonstrates how cultural frameworks determine which component of bias (ingroup love vs. outgroup hate) is most labile—individualistic systems might enable reevaluation of ingroups, whereas collectivist systems might prioritize ingroup stability but allow outgroup attitude updating through novel positive experiences.

While our study identifies ingroup identification as a central driver of cultural differences in impression updating—particularly in the attenuation of ingroup favoritism—prior work underscores the distinct role of ingroup glorification (i.e., the belief in one’s group’s inherent superiority) in shaping intergroup dynamics. For instance, previous research demonstrated that glorification (but not mere identification) positively predicts outgroup derogation, suggesting it uniquely amplifies hostility toward external groups^57,58^. This raises the possibility that glorification may also modulate how outgroup impressions are revised, for example by resisting positive updating even in the face of disconfirming evidence. Future work should investigate whether glorification independently influences outgroup-specific learning processes, such as discounting positive outgroup interactions or amplifying negative ones.

Previous research in non-social domains identified different learning mechanisms from perceived losses and perceived gains. For example, participants showed faster learning-related changes in task performance if they had to minimize losses compared to seeking gains^41^. Outside the learning domains, ingroup bias manifested in the domain of gain appears to decrease in the domain of losses when people act as decision-makers^44,45^. Differential learning effects in the gain and the loss domain may be related to a higher sensitivity to losses compared to gains^52,59^, and converge with evidence showing that inflicting losses (i.e., taking from someone) is considered as socially less appropriate than providing gains (i.e., giving to someone)^60^. Extending this literature, we investigate the effect of Gain and Loss frames on intergroup impression formation and demonstrate that the learning-related decrease of ingroup bias is stronger in loss compared to gain domains.

Previous research on impression formation in intergroup settings provided mixed findings. While some found a decrease in ingroup bias after having objectively same experiences with ingroup and outgroup members^34,50^, others found that ingroup bias remained intact as negative experiences with outgroup members have a more substantial impact on impression updating than similar experiences with ingroup members^24,36,61^. Our study provides a possible explanation for this incongruency and highlights the importance of the perceived valence of ingroup and outgroup interactions (i.e., positive/gain versus negative/loss) as well as individual differences in ingroup identification.

The findings of this study also have practical implications for promoting intergroup harmony and reducing ingroup bias. For example, our results suggest that framing ingroup experiences as losses rather than gains can effectively diminish ingroup bias by helping us see the ingroup in a more objective light. In addition, gains from the outgroup can contribute to improving outgroup impressions. The observed cross-cultural consistency in this learning-related bias reduction suggests that such strategies could be applicable across different cultural contexts. Apart from that, our findings also highlight the importance of considering individual differences, such as the degree of ingroup identification, which may influence the effectiveness of these interventions.

Our two independent samples consisted of male participants. This allowed us to control for unspecific gender effects (e.g., induced by gender-mixed pairings of participants and confederates), and gender differences in the expression of ingroup favoritism^62^ but limited the generalizability of current results. Future research should investigate the contextual influence on intergroup impression formation in females. Although our findings demonstrate consistent results across both studies, the differing group induction procedures used in each study may introduce context-specific confounding factors. Future research could improve comparability by adopting standardized induction protocols, thereby strengthening methodological consistency and reducing potential contextual biases.

In conclusion, our results demonstrated that negative ingroup experiences contribute to decreases in ingroup bias in the loss, but not in the gain domain. This effect was common in individuals from Western societies and limited to individuals with weak ingroup identification in East Asian societies. These findings highlight the importance of context (here Gain or Loss frames) in social learning and show the value of cross-cultural research for uncovering mediating variables.

## Methods

### Participants

We recruited two independent samples for the two studies.

#### Study 1 - Western sample

112 healthy Swiss males (58 in the Gain frame, mean age ± SD = 24.0 ± 2.56 years) participated in Study 1 as paid volunteers. We chose an all-male instead of a gender-mixed group of participants so that we could also use all-male confederates and avoid the complications of the gender-mixed pairing of participants and confederates (see Group induction in pre-session in *supplementary methods*).

#### Study 2 - East Asian sample

100 healthy Chinese males (50 in the Gain frame, mean age ± SD = 21.50 ± 2.66 years) participated in Study 2 as paid volunteers. One participant in the Loss frame was excluded because of technical issues during the experiment. Thus, data from 99 participants were analyzed.

Study 1 was approved by the Research Ethics Committee of the Canton (state) of Zurich, and Study 2 was approved by the local Research Ethics Committee at Peking University, China. Both studies were conducted in accordance with the Declaration of Helsinki. All participants had normal or corrected-to-normal vision, no history of psychological or neurological disorders, and provided written informed consent after the experimental procedure had been fully explained. Participants were reminded of their right to withdraw at any time during the study.

A sensitivity analysis using G*Power 3.1 indicates that given α (5%) and considering within-between interactions with two groups (frame: gain, loss) and four conditions (time (before/after learning)×group (ingroup/outgroup)), a sample size of *N* = 112 (Study 1) has 80% power to detect a small to medium effect size of *f* = 0.11. The sample size for Study 2 was determined by an a priori power analysis using G*Power 3.1^63^ for a within-between interaction in a repeated-measures analysis of variances (ANOVA) design with two between-subject (frame: gain, loss) and four within-subject conditions (time (before/after learning)×group (ingroup/outgroup)). A total sample size of 90 participants (45 participants per group) was required for each study to detect a small to medium effect size of *f* = 0.21 (as calculated by the effect size of three-way interaction in Study 1) at *α* = 0.05 (two-tailed) with a power of 80%. We recruited more than 90 participants to account for possible data loss (10%).

### Group induction in pre-session

Before starting the learning task, participants underwent a group induction procedure.

In both studies, social group induction was based on nationalities. Participants were told that the experiment aimed to examine cultural differences in decision-making. In Study 1, we recruited Swiss participants and welcomed them together with six other Swiss participants (ingroup) and six Middle Eastern confederates (outgroup). Participants and confederates briefly met in the waiting area but did not interact. They were assigned a number ranging from 1 to 13 which determined their place in the testing lab. The testing lab consisted of two rooms that could be separated by a sliding door. Care was taken to ensure that all Western participants and all Middle Eastern confederates were placed in different partitions. The separated rooms were named “Room A” and “Room B”, and the relationships between room name and group membership were counterbalanced across participants.

In Study 2, we recruited Chinese participants who were shown prerecorded choices from one of six other Chinese participants (ingroup) or one of six German participants (outgroup). The observed rating originated from “Room A” or “Room B”, indicating the nationality of the other player, counterbalanced across participants.

A well-established priming procedure^64^ was used to enhance the respective social group manipulations. Specifically, participants were asked to write down five attributes that are typical for Middle Eastern males and Swiss males (Study 1) or German males and Chinese males (Study 2). This procedure is commonly used to activate group-related stereotypes^50^, in our case to activate the stereotype of Middle Eastern males in Western participants or the stereotype of German males in East Asian participants.

To avoid possible reputation effects that might influence participants’ behavior, all ratings were kept anonymous, and we ensured that the participants did not meet the confederates after the experiment in Study 1. Thus, all ratings and decisions were personal and could not be observed by the other participants.

### Experimental procedure of learning task

Each trial began with a fixation cross displayed for 1–7 s. Next, two closeness rating scales were presented for 5 s each. Participants used a manikin to indicate their perceived closeness to individuals in Room A and Room B by moving it along a 10-step scale. The manikin’s starting position was randomized to keep participants engaged. The order of closeness ratings for Rooms A and B was randomized across trials. We selected the social closeness scale based on our prior finding that social interactions with both ingroup and outgroup members consistently influenced participants’ perceived social closeness towards them^34^. This measure is particularly relevant as heightened closeness strengthens prosocial intentions^65,66^. Furthermore, reporting social distance may reduce social desirability concerns compared to ratings of unpleasantness or warmth.

After the closeness ratings, a letter was shown for 2 s, indicating the room where the other player (i.e., the person who could ostensibly give/take money to/from the participants) was located. Participants then indicated their expectancy of receiving 5 MU (vs. 0 MU, Gain frame) or losing 5 MU (vs. 10 MU, Loss frame) from/to the other person in the current trial using a 10-step rating scale. In the Gain frame, participants received an initial endowment of 0 monetary units (MU), and the ingroup or outgroup individual could give 5 or 0 MU (Fig. 1a). In the Loss frame, participants received an initial endowment of 10 MU, and the ingroup or outgroup individual could take 5 or 10 MU (Fig. 1b). The two frames were mathematically equivalent, yielding identical final payoff states.

The decision of the other individual was then shown for 2 s. In the Gain frame, a 5 MU symbol indicated a gain of 5 MU, while a 0 MU symbol indicated no gain. In the Loss frame, a 5 MU symbol indicated a loss of only 5 MU, leaving the participant with 5 MU, while a 10 MU symbol indicated a loss of all 10 MU, leaving the participant with 0 MU. The feedback participants received (e.g., whether they gained or lost money, ostensibly due to an ingroup/outgroup member’s decision) was algorithmically generated by a computer program, not by actual participants. This design ensured that every participant experienced identical feedback sequences, regardless of group assignments. Participants experienced positive events in 75% of all trials in both frames, with equalized and primarily positive objective experiences with both ingroup and outgroup conditions. The learning experiment consisted of four blocks of 24 trials each, totaling 96 trials.

### Questionnaires

We used an impression scale to assess participants’ impressions towards ingroup (Study 1/Study 2: Swiss/Chinese) and outgroup (Study 1/Study 2: Middle Eastern/German) individuals before and after the learning experiment, similar to our previous research^34^. Moreover, participants completed the ingroup identification questionnaire^67^, which measures overall identification and satisfaction with the ingroup. In Study 1, Western participants also indicated their tendency for outgroup discrimination using the Modern Racism Scale, modified to the Middle Eastern context^68^, as validated in previous research^34^. We did not employ the Modern Racism Scale in Study 2 because a validated Chinese translation was not available. The impression ratings of 15 participants in Study 1 and one participant in Study 2 were missing and replaced by the average impression rating of all other participants in the corresponding frame. Using the impression rating without replacement yields similar results.

### Analyses of impression, expectancy, and closeness ratings

We used linear mixed models (LMM, ‘lme4’, Bates et al., 2015) in R-4.3.2 for the behavioral analyses on impression, expectancy, and closeness ratings. The expectancy ratings correspond to participants’ subjective probability estimate of receiving a positive outcome. Higher expectancy ratings correspond to a higher subjective probability of receiving a positive outcome from others.

First, as a manipulation check, we examined the difference between ingroup and outgroup across multiple measurements at the beginning of the experiment. Specifically, we ran LMMs with group (ingroup/outgroup), frame (Gain frame/Loss frame), and culture (Swiss/Chinese) and all interactions as predictors, and the impression (acquired before learning experiment), expectancy (the first trial) and closeness (the first trial) ratings as the dependent variable. We used participants as random intercepts and by-participant random slopes for the fixed effects of the group.

We then examined the learning-related impression changes and compared them between frames. To this end, we ran LMMs with group (ingroup/outgroup), time (before/after learning), frame (gain/loss), and all the interactions as predictors, and the impression rating scores as the dependent variable. We used participants as random intercepts and by-participant random slopes for the fixed effects of group and time. The Anova() function from the car package served to assess the significance of the fixed effects.

### Computational modeling

First, we modeled participants’ trial-by-trial expectancy ratings using a Rescorla-Wagner reinforcement-learning model (Rescorla & Wagner, 1972) to test for differences in basic learning mechanisms between the ingroup and outgroup conditions. The RL model assumes that participants change their expectancy of receiving an outcome when new information reveals that the experienced outcome differs from the expected outcome.

1 $${{V}_{i}\left(t+1\right)=V}_{i}\left(t\right)+{{\alpha }_{i}\delta }_{i}$$

2 $${\delta }_{i}\left(t\right)=R\left(t\right)-{V}_{i}\left(t\right)$$

On each trial *t*, the value of the (future) expectation ${V}_{i}\left(t+1\right)$ for group *i* (ingroup or outgroup) is a function of the current value ${V}_{i}\left(t\right)$ and the prediction error ${\delta }_{i}$ (Eq. 1), which corresponds to the difference between the experienced outcome *R(t)* at trial *t* (coded as 0 or 1 for negative or positive events) and the value of the current expectation *V*_*i*_ (Eq. 2). The learning rate α (0 ≤ *α* ≤ 1) controls the extent to which the current expectation is updated by new information. Consequently, a low learning rate corresponds to a slow integration of prediction errors into current values. To account for individual differences in prior expectations, the initial expectancy rating for both the ingroup and outgroup was set equal to each participant’s actual expectancy rating for those groups during the first trial. Finally, the current value was multiplied by a response parameter *β* (*β* > 0), which linearly mapped the current value to expectancy ratings (Eq. 3).

3 $$Expectancy\,Ratin{g}_{{\rm{i}}}\,{=\beta \times V}_{i}\left(t\right)$$

We fitted participants’ expectancy ratings to several models, including models that assumed the same learning rate for the ingroup and the outgroup (Learning Model 1), different learning rates for the two groups (Learning Model 2), and different learning rates for positive and negative prediction errors generated by the ingroup or outgroup (Learning Model 3), resulting in three models tested in total. We identified the maximum likelihood estimate using least squares to fit the model.

Next, we fitted the trial-by-trial closeness ratings regarding the ingroup or outgroup as a linear function of previous prediction errors, i.e., based on the winning model determined above^34^. We assume that the closeness ratings are influenced by the prediction errors based on the following reasons: First, in our previous study (Zhou, Lindström, et al., 2022), which employed a similar experimental design, we successfully modeled closeness ratings using prediction errors. The model demonstrated a good fit, and the derived weight parameter was correlated with post-learning changes in intergroup impressions. Second, we performed an additional analysis in which we extracted trial-wise prediction errors and examined their association with subsequent changes in closeness ratings. It revealed a statistically significant relationship between prediction errors and closeness updates across both Chinese and Swiss participants (ps < 0.001, Supplementary Fig. 4), further supporting the association between closeness updates and trial-wise prediction errors.

In all models, we assumed that changes in closeness to group *i* (i.e., the ingroup or the outgroup) were a linear function of the time-discounted sum of previous prediction errors to outcomes from *i* (as originating from the RL model, Eqs. 1–3)

4 $$\varDelta {{Closeness}}_{i}\left(t\right)={W0+W}_{i}\mathop{\sum }\limits_{j=1}^{t}{\gamma }^{t-j}{\delta }_{{ij}}$$

5 $$\varDelta {{Closeness}}_{i}\left(t\right)={W0+W}_{{pos}}\mathop{\sum }\limits_{j=1}^{t}{\gamma }^{t-j}{\delta {pos}}_{{ij}}+{W}_{{neg}}\mathop{\sum }\limits_{j=1}^{t}{\gamma }^{t-j}{\delta {neg}}_{{ij}}$$

Closeness Model 1 (Eq. 4) assumes that closeness updates to group *i* result from previous prediction errors to outcomes arising from group *i*. Closeness Model 2 (Eq. 5) is similar to Closeness Model 1, except that it separates the prediction errors into positive and negative ones and adds them up separately. If trial *t* entails a decision from an outgroup individual, the closeness updates for the ingroup at that trial result from the time-discounted sum of the previous ingroup-related prediction errors plus an additional prediction error of zero, which means that the decision from outgroup/ingroup would not influence the closeness updates for ingroup/outgroup. This winning model (Closeness Model 2) has three parameters. The parameter *W*_*pos*_*/W*_*neg*_ captures the magnitude (weight) of the influence of positive/negative prediction errors on changes in closeness. The *W*-parameter ranges from -10 to 10, where 10 represents the maximum of the closeness ratings. A larger *W* corresponds to a stronger influence of prediction errors on closeness updates. The parameter *γ* (0 ≤ *γ* ≤ 1) captures an exponential decay of the influence of previous prediction errors over time, such that the more recent prediction errors have a greater impact on the changes in closeness than the earlier prediction errors. If *γ* is close to one, all preceding prediction errors receive the same weight, and if it is close to zero, only the last prediction error leads to subsequent changes in closeness.

### Parameter estimation and model comparisons

To fit the parameters of the different computational models, we identified the maximum likelihood estimate using least squares, which determines the set of parameters that maximize the probability of trial-by-trial expectancy ratings or closeness updates given the specific model. Both the Learning Models and Closeness Models were estimated based on a Gaussian likelihood function. Parameters were independently fitted in each participant using the Broyden-Fletcher-Goldfarb-Shanno (BFGS) optimization method. To avoid local minima in parameter fitting, optimization was initiated with randomly selected start values^69^. Model implementations and parameter fitting were done in R v.4.3.2 (R Development Core Team, 2012). To examine the degree to which the model explained the experimental data, we also calculated the mean squared error over the expectancy and closeness ratings.

For model comparison, we computed the Akaike Information Criterion (AIC), which penalizes the model evidence with model complexity as follows: AIC = -2ln(L) + 2k, where –ln(L) is the negative log-likelihood, and k is the number of model parameters. The individual model comparison criteria (AIC) were then fed to the mbb-vb-toolbox (https://code.google.com/p/mbb-vb-toolbox/). This toolbox calculates the exceedance probability (denoted as XP) for each model within the set of models, helping us select the best model. An XP > 95% for one model within a set is typically considered as significant evidence in favor of this model being the most likely.

### Model identification

We used simulations to assess that our experiment allowed us to dissociate models of interest, as well as identify parameters of interest within the winning model. We performed a model identifiability analysis by simulating data from 30 participants and fitting this simulated dataset to all the candidate models^70^. We used the same estimation method as applied to the experimental participants’ data and repeated the whole procedure ten times. For the learning model, α was varied from 0 to 1, and the β parameters were varied from 0 to 3 (the range shown by our participants). For the closeness model, we set the *W* parameters from -10 to 10, *W0* parameters from 0 to 10, and the γ parameters from 0 to 1. By plotting the confusion matrices of how many times each model won (based on AIC measures), we show that the models are identifiable with our model comparison process.

### Parameter recovery

To assess the reliability of the winning model and the interpretability of the free parameters, we also performed parameter recovery on simulated data as recommended for modeling analyses^70,71^. To do this, we computed the Pearson correlation between parameter values that were used for generating simulated data and the values recovered by the model fitting procedure. A high correlation indicates a reliable parameter fitting procedure.

In more detail, we simulated the data 100 times and used the same estimation method as applied to the experimental participants’ data to estimate the parameters. We performed the parameter recovery analyses on the winning models. Similar to the model identification analyses, for the learning model, we varied *α* from 0 to 1, and bounded the *β* parameters at 0 and 3 to reflect the range shown by our participants. For the closeness model, we set the *Wpos/Wneg* parameters from -10 to 10, *W0* parameters from 0 to 10, and set the *γ* parameters from 0 to 1. For both reinforcement learning models and Closeness models, we found high Pearson’s correlations between the true (simulated) and the estimated parameter values (learning model: *rs* > 0.88; closeness model: *rs* > 0.98), suggesting that our parameter fitting procedure was reliable.

### Analyses of modeling parameters

We ran LMM to analyze the learning rates and weight parameters. Specifically, LMMs used group (ingroup/outgroup), valence (positive/negative), frame (gain/loss), and their interactions as predictors, and the learning rate/weight parameters as the dependent variable. Participants served as random intercepts and slopes for the fixed effects of group and valence.

We performed multiple linear regression analyses to determine whether learning mechanisms—quantified via computational parameters—drive changes in intergroup impressions in the Gain frame and Loss frame, respectively. Specifically, we entered all the parameters related to learning (i.e., the learning rates and the weight parameters) into the model that involved forward and backward stepwise regressions. The learning rate (α) parameter quantifies the degree to which participants update their expectations in response to prediction errors whereas the weight parameter (w) represents the extent to which prediction errors influence updates in social closeness. Then, we entered the change of intergroup impression as the dependent variable. Stepwise regression is a data-driven method to determine the significant predictors in linear regression models. After the selection of the significant predictors, we tested whether the significance was robust to the removal of outliers and influential cases. The latter was identified with the *influence measures* function in R (see^72^ for a similar approach). We only report significant results in both cases, i.e., using the whole data set as well as after removing influential cases.

## Supplementary information

Supplementary information

## Data Availability

The data that support the findings of the current study are available at https://osf.io/cbpm4/.

## Code availability

The codes that support the findings of the current study are available at https://osf.io/cbpm4/.
