# Supplementary material for: Learning reduces ingroup bias more with perceived losses than gains across cultures
Source: NPJ Sci Learn. 2025 Nov 10;10:76. doi: 10.1038/s41539-025-00362-x (PMC12603063; doi:10.1038/s41539-025-00362-x)
Supplement: Supplementary file 1 — Supplementary information [file 41539_2025_362_MOESM1_ESM.pdf]

Supporting Information for

Learning Reduces Ingroup Bias More with Perceived Losses than Gains Across Cultures

Yuqing Zhou[1 2+*], Björn Lindström[3+], Alexander Soutschek[4], Pyungwon Kang[5], Shihui Han[6], Philippe N. Tobler[5#] & Grit Hein[2#]

[1] State Key Laboratory of Cognitive Science and Mental Health, Institute of Psychology, Chinese Academy of Sciences, Beijing, China
[2] Translational Social Neuroscience Unit, Department of Psychiatry, Psychosomatics, and Psychotherapy, University of Würzburg, Würzburg, Germany
[3] Karolinska Institutet, Department of Clinical Neuroscience, Solna, Sweden.
[4] Department of Psychology, Ludwig Maximilian University, Munich 80802, Germany,
[5] Department of Economics and Laboratory for Social and Neural Systems Research, University of Zurich and Neuroscience Center Zurich, University of Zurich and Swiss Federal Institute of Technology Zurich, Zurich, Switzerland
[6] School of Psychological and Cognitive Sciences, Beijing Key Laboratory of Behavior and Mental Health, PKU-IDG/McGovern Institute for Brain Research, Peking University, Beijing, China

+ contributed equally to this work
# shared senior authorship

Address correspondence to:
Dr. Yuqing Zhou, zhouyq@psych.ac.cn

This PDF file includes:

Supplementary results
Learning rates analyses in East Asian sample (Study 2)

Supplementary Figure 1~4
Supplementary Table 1~7

Supplementary results

Learning rates analyses in East Asian sample (Study 2)

Unlike Study 1, the learning rate in Study 2 showed a significant group × valence interaction effect ($\chi 2$ = 5.20, p = 0.023, **Supplementary Tables 6 and 7**). Specifically, the learning rate for negative prediction errors was lower for the ingroup than the outgroup (M (ingroup vs. outgroup) = 0.10 vs. 0.15, $\chi 2$ = 7.29, p = 0.007, **Supplementary Figure 3**). In contrast, the learning rate for positive prediction error did not show differences between groups (M (ingroup vs. outgroup) = 0.46 vs. 0.40, $\chi 2$ = 1.50, p = 0.22, **Supplementary Figure 3**). We therefore compared the learning rate between cultures. The between studies comparison revealed that the differential learning rates between ingroup and outgroup for negative (i.e., α_{neg_in} - α_{neg_out}) prediction errors were significantly lower for East Asian than Western participants (t(209) = -2.5, p = 0.014), while the learning rates difference for positive (i.e., α_{pos_in} – α_{pos_out}) prediction errors were similar for East Asian and Western participants (t(209) = 0.91, p = 0.37).

Given that the cultural group showed differences in ingroup identification scores, we tested whether individual differences in ingroup identification scores could explain cultural differences in learning rates using a standard mediation model. To this end, we first determined the differences in learning rates between ingroup and outgroup for negative (i.e., α_{neg_in} - α_{neg_out}) and positive (i.e., α_{pos_in} – α_{pos_out}) prediction errors. Then, we used these differences as dependent variables, culture (Western/East Asian) as the independent variable, and the ingroup identification scores as the mediator. The

results first revealed that culture was associated with ingroup identification as indicated by lower ingroup identification scores for Western than East Asian participants (β = -0.53, $p < 0.001$). Moreover, the ingroup identification scores were negatively associated with the learning rate difference for negative prediction errors (β = -0.23, p = 0.007) but positively associated with the learning rate difference for positive prediction errors (β = 0.19, p = 0.02). Thus, the more strongly individuals identified with their ingroup, the less negative was their learning rate for the ingroup (vs. outgroup) but the more positive was their learning rate for the ingroup (vs. outgroup). Most importantly, the ingroup identification scores also significantly mediated the relationship between culture and ingroup bias in learning rates for negative (standardized indirect effect = 0.12, 95% CI [0.023, 0.225], **Supplementary Figure 3**) and positive (standardized indirect effect = -0.10, 95% CI [-0.18 -0.01], **Supplementary Figure 3**) prediction errors. The results suggest that ingroup identification is a potential intermediate mechanism underlying the associations between culture and group-related differences in learning mechanism

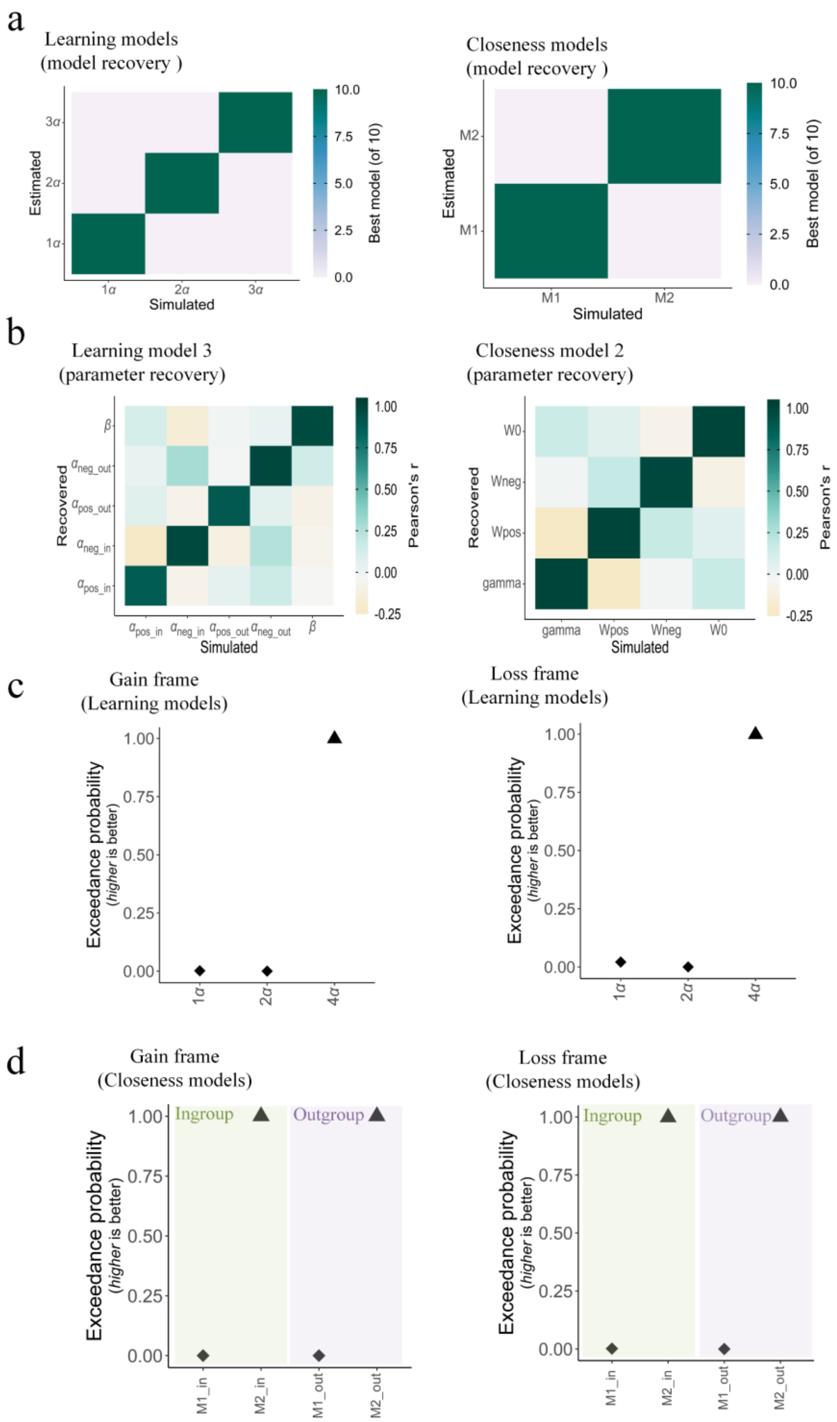

Supplementary Figure 1. Model diagnostics and comparison. (a) Model identification analyses for Learning models and Closeness models. Data were simulated 30 times with each candidate model. Then, AIC served to determine the winning model. This procedure was repeated 10 times, and the confusion matrices showed how many times each model won. Strong diagonals represent reliable model identifiability. (b) Parameter recovery was performed on data simulated by the winning learning model (Learning Model 3) and the winning closeness model (Closeness Model 2). The confusion matrix represents correlations between simulated and fitted parameters. Dark colors on the diagonal indicate that parameters can be recovered. (c) Learning Model

3 (4α, four learning rates model, triangle) was the best model based on exceedance probability for both the Gain and Loss frame. (d) Closeness model 2 (triangle) was the best model based on exceedance probability for both the Gain and Loss frame and ingroup and outgroup closeness ratings.

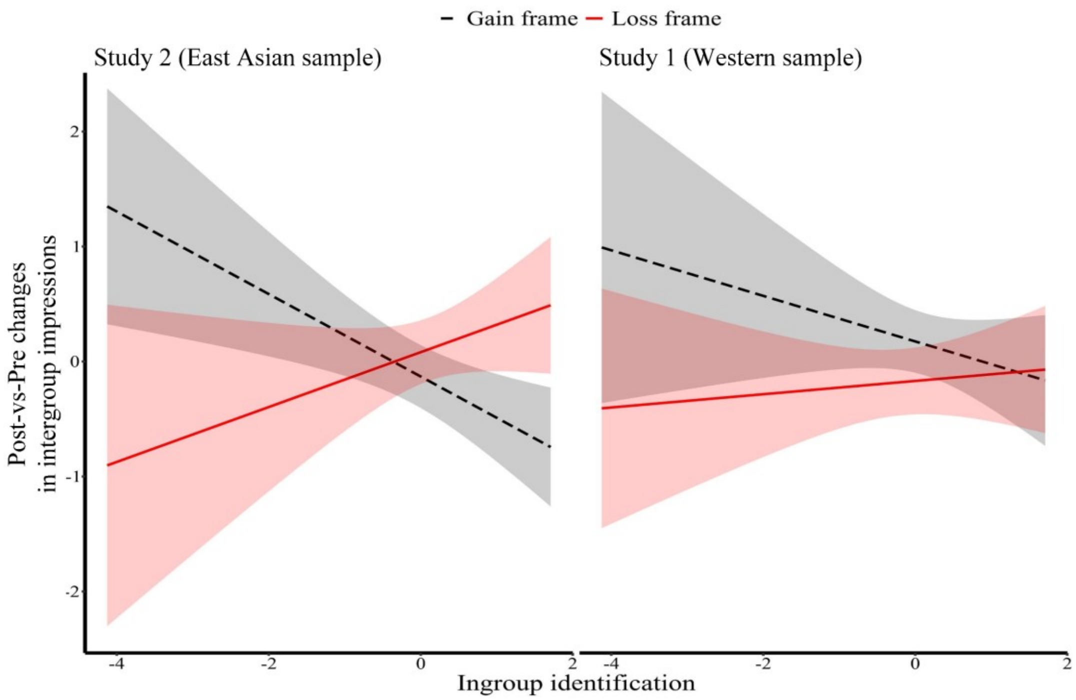

Supplementary Figure 2. Testing the modulating effect of ingroup identification scores on impression changes for East Asian (Study 2, left panel) and Western participants (Study 1, right panel). The ingroup identification × frame pattern was similar for the East Asian and Western samples (β = -0.34, $t(187)$ = -1.18, p = 0.24).

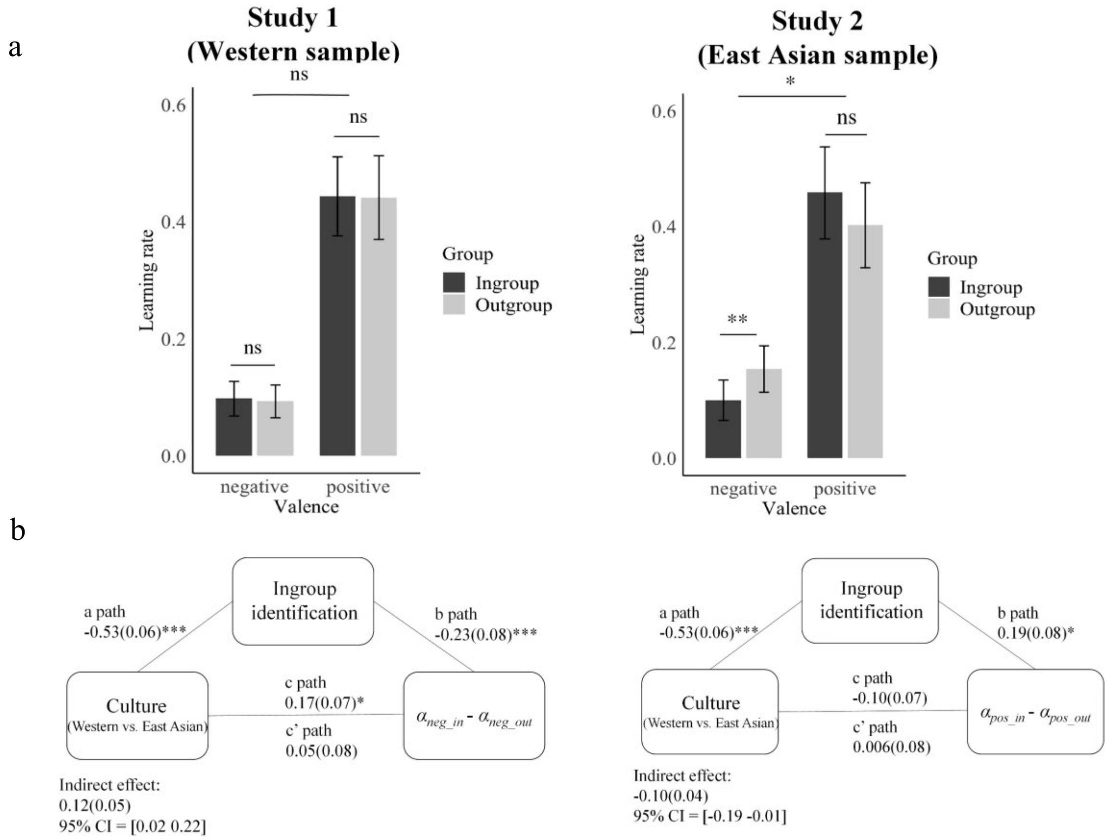

Supplementary Figure 3. Analyses of Learning Rates (a) Unlike in the Western sample ($\chi2$ = 0.00, p = 0.95), the Group × Valence interaction was significant in the East Asian sample ($\chi2$ = 5.20, p = 0.023). (b) Ingroup identification mediating the effect of culture on group bias. Ingroup identification scores mediated the relationship between culture and group bias in learning rates for both negative (standardized indirect effect = 0.12, 95% CI [0.023, 0.225]) and positive prediction errors (standardized indirect effect = -0.10, 95% CI [-0.18 -0.01]).

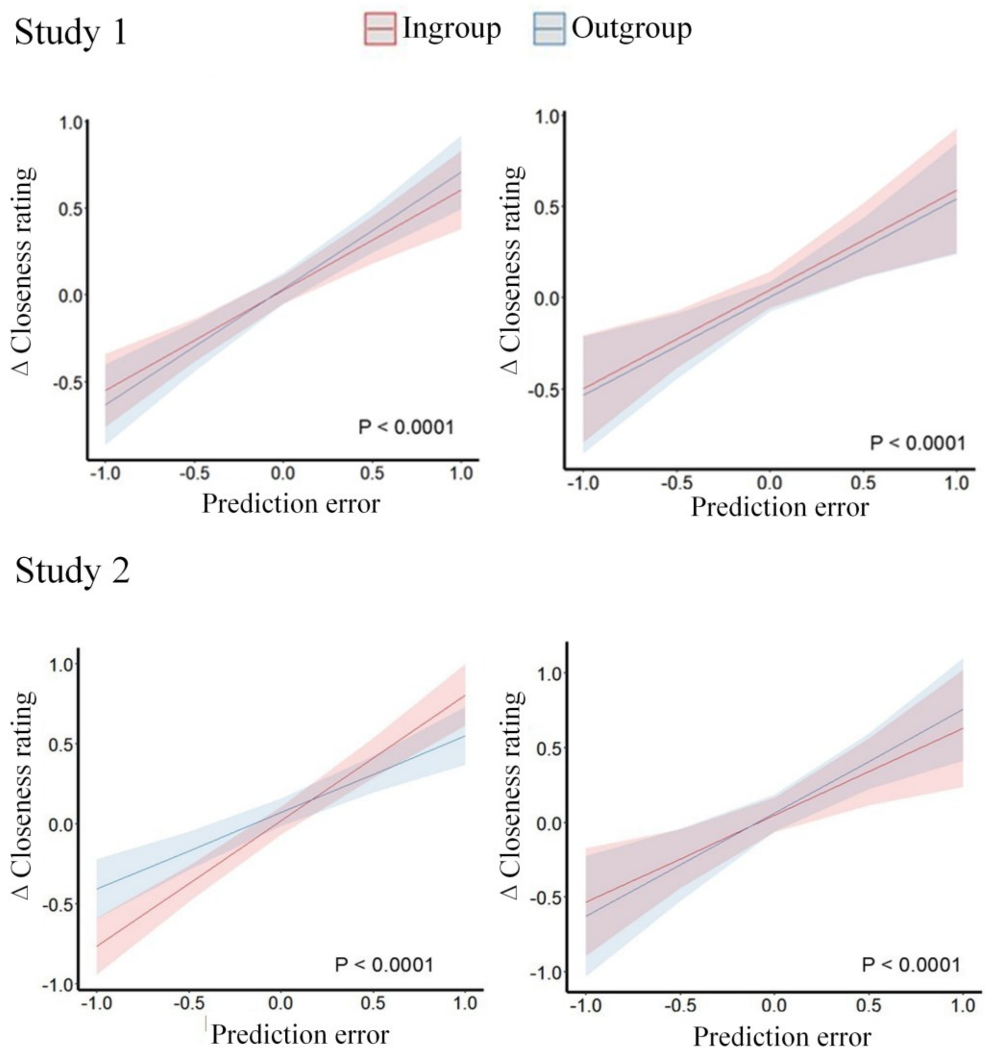

Supplementary Figure 4. The positive association between trial-wise prediction error and closeness updates across studies.

Supplementary Table 1. Results of linear mixed models examining the ingroup bias in impression, expectancy, and closeness ratings at the beginning of the task in both samples (Studies 1 and 2 combined)

**Dependent variables**	**Regressors**	**Statistic value** χ^2	*p*
**Impression ratings**	Frame	1.10	0.295
	**Group**	**176.28**	**< 0.001**
	Culture	0.07	0.798
	Frame × Group	1.07	0.300
	**Frame × Culture**	**4.37**	**0.037**
	**Group × Culture**	**83.39**	**< 0.001**
	Frame × Group × Culture	0.70	0.403
**Expectancy ratings**	Frame	2.13	0.145
	**Group**	**9.20**	**0.002**
	**Culture**	**6.29**	**0.012**
	Frame × Group	1.62	0.203
	Frame × Culture	1.12	0.289
	Group × Culture	1.48	0.224
	Frame × Group ×Culture	0.02	0.881
**Closeness ratings**	Frame	3.28	0.07
	**Group**	**45.11**	**< 0.001**
	**Culture**	**12.52**	**< 0.001**
	Frame × Group	1.07	0.300
	**Frame × Culture**	**4.97**	**0.026**
	**Group × Culture**	**92.36**	**< 0.001**
	Frame × Group × Culture	0.62	0.431

Supplementary Table 2. Computational model parameters of the winning model in the Western sample (Study 1)

**Computational Parameters**	**Loss frame Mean (SD)**	**Gain frame Mean (SD)**
Weight on positive prediction error from ingroup (W_{pos_in})	0.88 (2.42)	1.44 (2.89)
Weight on positive prediction error from outgroup (W_{pos_out})	0.25 (2.06)	0.89 (2.00)
Weight on negative prediction error from ingroup (W_{neg_in})	0.38 (1.20)	0.56 (0.93)
Weight on negative prediction error from outgroup (W_{neg_out})	0.60 (2.38)	0.46 (1.35)
Baseline closeness for ingroup ($W0_{in}$)	4.92 (2.56)	4.13 (2.36)
Baseline closeness for outgroup ($W0_{out}$)	5.47 (2.45)	4.27 (2.38)
Discount factor for ingroup ($gamm_{in}$)	0.82 (0.30)	1.44 (2.89)
Discount factor for outgroup ($gamm_{out}$)	0.82 (0.29)	0.80 (0.31)
Learning rate for positive prediction errors from ingroup (α_{pos_in})	0.47 (0.36)	0.42 (0.36)
Learning rate for positive prediction errors from outgroup (α_{pos_out})	0.49 (0.40)	0.40 (0.37)
Learning rate for negative prediction errors from ingroup (α_{neg_in})	0.12 (0.19)	0.08 (0.12)
Learning rate for negative prediction errors from outgroup (α_{neg_out})	0.10 (0.17)	0.09 (0.12)
Response parameter (β)	1.05 (0.52)	1.16 (0.56)

Supplementary Table 3. Results of a linear mixed model testing the effects of group (ingroup/outgroup), frame (Gain frame/Loss frame), and valence (positive/negative) on learning rates and weight parameters in the Western sample (Study 1)

		Statistic value	
**Parameters**	**Regressors**	χ^2	*P*
**Learning rate (α)**	Frame	2.23	0.14
	Group	0.03	0.85
	Valence	**112.97**	**< 0.001**
	Frame × Group	0.01	0.91
	Frame ×Valence	0.49	0.49
	Group ×Valence	0.00	0.95
	Frame × Group ×Valence	0.59	0.44
**Weight *(W)***	Frame	1.50	0.22
	Group	2.90	0.088
	Valence	3.58	0.059
	Frame × Group	0.13	0.72
	Frame ×Valence	2.26	0.13
	Group ×Valence	**6.12**	**0.013**
	Frame × Group ×Valence	0.59	0.44

Supplementary Table 4. Comparisons of model fit for expectancy ratings in East Asian sample (Study 2)

		Loss frame			Gain frame		
**Computational models**	**K**	**Mean r^2**	**AIC**	**XP**	**Mean r^2**	**AIC**	**XP**
**Expectancy ratings**							
One learning rate	2	0.18	-14684	0	0.22	-16330	0
Two learning rates	3	0.22	-14598	0	0.26	-15720	0
**Four learning rates**	**5**	**0.29**	**-16010**	**1**	**0.32**	**-17776**	**1**

Supplementary Table 5. Comparisons of model fit for closeness ratings in East Asian sample (Study 2)

		Loss frame			Gain frame		
**Computational models**	**K**	**Mean r^2**	**AIC**	**XP**	**Mean r^2**	**AIC**	**XP**
**Ingroup closeness ratings**							
Group-specific prediction error weights	3	0.13	1760	0.02	0.15	-770	0
Outcome-specific prediction error weights	**4**	**0.19**	**1492**	**0.98**	**0.23**	**-1078**	**1**
**Outgroup closeness ratings**							
Group-specific prediction error weights	3	0.11	2585	0	0.11	2251	0
Outcome-specific prediction error weights	**4**	**0.20**	**1878**	**1**	**0.26**	**1186**	**1**

Supplementary Table 6. Computational model parameters of the winning model in East Asian sample (Study 2)

**Computational Parameters**	**Loss frame**	**Gain frame**
	**Mean (SD)**	**Mean (SD)**
Weight on positive prediction error from ingroup (W_{pos_in})	0.36 (1.57)	0.24 (2.10)
Weight on positive prediction error from outgroup (W_{pos_out})	0.56 (1.79)	0.96 (1.73)
Weight on negative prediction error from ingroup (W_{neg_in})	0.39 (1.33)	0.38 (1.47)
Weight on negative prediction error from outgroup (W_{neg_out})	0.30 (1.50)	0.34 (0.72)
Baseline closeness for ingroup ($W0_{in}$)	6.58 (1.78)	7.07 (2.09)
Baseline closeness for outgroup ($W0_{out}$)	4.67 (2.12)	4.75 (2.42)
Discount factor for ingroup ($gamm_{in}$)	0.81 (0.28)	0.73 (0.38)
Discount factor for outgroup ($gamm_{out}$)	0.88 (0.20)	0.88 (0.20)
Learning rate for positive prediction errors from ingroup (α_{pos_in})	0.46 (0.39)	0.93 (0.19)

Learning rate for positive prediction errors from outgroup (α_{pos_out})	0.43 (0.39)	0.37 (0.34)
Learning rate for negative prediction errors from ingroup (α_{neg_in})	0.11 (0.18)	0.10 (0.17)
Learning rate for negative prediction errors from outgroup (α_{neg_out})	0.17 (0.23)	0.14 (0.18)
Response parameter (β)	0.80 (0.36)	0.87 (0.35)

Supplementary Table 7. Results of a linear mixed model testing the effects of group (ingroup/outgroup), frame (Gain frame/Loss frame), valence (positive/negative) on learning rates and weight parameters in East Asian sample (Study 2)

		**Statistic value**	
**Parameters**	**Regressors**	χ^2	*P*
**Learning rate (α)**	Frame	0.68	0.41
	Group	0.00	0.97
	Valence	**65.80**	**< 0.001**
	Frame × Group	0.54	0.46
	Frame ×Valence	0.01	0.91
	Group ×Valence	**5.20**	**0.023**
	Frame × Group ×Valence	0.29	0.59
**Weight (*W*)**	Frame	0.17	0.68
	Group	1.83	0.18
	Valence	1.30	0.25
	Frame × Group	0.94	0.33
	Frame ×Valence	0.18	0.67
	Group ×Valence	3.59	0.058
	Frame × Group ×Valence	0.71	0.400
